# Iodine Excretion in 24-hour Urine Collection and Its Dietary Determinants in Healthy Japanese Adults

**DOI:** 10.2188/jea.JE20150245

**Published:** 2016-12-05

**Authors:** Ryoko Katagiri, Keiko Asakura, Ken Uechi, Shizuko Masayasu, Satoshi Sasaki

**Affiliations:** 1 Department of Social and Preventive Epidemiology, Graduate School of Medicine, University of Tokyo, Tokyo, Japan; 2 Department of Social and Preventive Epidemiology, School of Public Health, University of Tokyo, Tokyo, Japan; 3 Interfaculty Initiative in Information Studies, University of Tokyo, Tokyo, Japan; 4 Ikurien-naka, Naka, Ibaraki, Japan

**Keywords:** iodine, urinary excretion, Japanese, dietary determinants, 24-hour urine collection

## Abstract

**Background:**

Since seaweed is a common component of the Japanese diet, iodine intake in Japanese is expected to be high. However, urinary iodine excretion, measured using 24-hour urine samples, and its dietary determinants are not known.

**Methods:**

Apparently healthy adults aged 20 to 69 years living in 20 areas throughout Japan were recruited in February and March, 2013. Urinary iodine excretion was evaluated using 24-hour urine collected from 713 subjects (362 men and 351 women), and the difference among age groups was assessed. The association between dietary intake of food groups and urinary iodine excretion was assessed among 358 subjects who completed a semi-weighed 4-day diet record (DR) and urine collection. The correlations between iodine intake and iodine excretion were also evaluated, and correlation coefficients were calculated for iodine intake in the DR of the overlapping day or the DR 1 day before and after urine collection.

**Results:**

Median iodine excretion in 24-hour urine was 365 µg, and excretion was significantly higher in older subjects. Iodine intake estimated by the DRs was significantly correlated with urinary iodine excretion when DRs and urine collection were obtained on the same day (*r* = 0.37). After adjustment for confounding factors, iodine excretion was significantly associated with intakes of kelp and soup stock from kelp and fish.

**Conclusions:**

Although multiple measurements for urinary iodine are required to confirm our results, this study showed the current iodine status of healthy Japanese adults. The results suggest that kelp and fish are the main contributors to Japanese iodine status measured by 24-hour urine.

## INTRODUCTION

Iodine deficiency disorders remain a public health target worldwide, and 35% of the world’s population is estimated to have insufficient iodine intake.^1^ In contrast, Japan is considered an iodine-replete area due to habitual seaweed consumption. A literature review of information based on diet records, urine iodine, and food surveys estimated that the iodine intake of Japanese was 1–3 mg/day.^2^

The World Health Organization (WHO) recommends urinary iodine concentration as a practical biomarker for iodine nutrition. To prevent iodine deficiency, the WHO has set a urinary iodine concentration of <100 µg/L as “Insufficient” and ≥300 µg/L as “Excessive” for people aged 6 years or older, other than pregnant and lactating women.^3^ 24-hour urinary iodine excretion is considered a better measure of iodine intake than urinary concentration from casual urine samples,^4^^,^^5^ although concentrations in morning spot urine samples from 37 Japanese subjects have been shown to be positively correlated with iodine excretion in 24-hour urine.^6^ Normally, almost all ingested iodine is considered to be absorbed, and up to around 90% is excreted in urine when intake is sufficient.^7^^–^^9^

Dietary iodine intake assessed with dietary records (DRs) or food frequency questionnaires (FFQs) has been associated with urinary iodine excretion in countries where seaweed consumption is uncommon^10^^,^^11^ and in Asian countries, where consumption is habitual.^12^ In Japan, however, the relationship between iodine intake as assessed with a validated dietary assessment method and 24-hour urinary iodine excretion has not been epidemiologically assessed. One study examined the association between an FFQ for iodine intake and urinary iodine concentration^13^ but used an FFQ that was validated for urinary iodine concentration only, and not for 24-hour urine excretion or DRs, which are both accepted gold standards for validation. Moreover, only a few studies have reported iodine intake assessed with a DR in Japan^14^ because, until the publication of the 2010 version of the “Standard Tables of Food Composition in Japan” (STFCJ), no iodine composition table was available.^15^ This composition table includes only 515 of 1878 food items in the STFCJ. Using the method of Rand et al,^16^ we developed a comprehensive iodine composition table,^17^ which covers 80% of food items in the STFCJ and enabled the estimation of iodine intake in Japanese from DRs.

Of note, younger Japanese consume less seaweed^18^ and might be at risk of iodine deficiency.^19^ In contrast, intake among older generations is relatively high,^20^ and some might extend beyond the tolerable upper intake level (UL) of the Dietary Reference Intakes (DRIs) of Japanese for 2015.^9^^,^^17^ Risk assessment for excess and deficiency diseases is currently difficult, however, because few studies have assessed iodine intake with urinary and clinical data. Furthermore, although several papers have reported urinary iodine status in Japanese, assessment was mainly done using spot urine, and those studies that did test 24-hour urine samples had a limited number of participants and study areas that were not nationally representative.^2^

Here, we collected 24-hour urine from over 700 participants in 20 areas throughout Japan. Our aim was to evaluate urinary iodine status in healthy Japanese adults from a nationwide survey, investigate the association between iodine intake and urinary iodine excretion, and to identify dietary determinants of iodine excretion.

## METHODS

### Study population

Apparently healthy men and women aged 20 to 69 years living in 20 areas throughout Japan were recruited in February and March, 2013. Details of the study have been reported elsewhere.^21^ Briefly, 10 dietitians working at 10 different welfare facilities in each of the 20 areas participated as research dietitians. The 20 study areas were selected based on geographical location and the feasibility of recruitment of research dieticians. In these 20 areas, 17 areas were individual prefectures and three areas were combinations of two prefectures each (Aomori and Iwate, Niigata and Toyama, and Fukuoka and Saga). One dietitian subsequently declined participation due to an accident, leaving 199 dietitians, who then recruited their co-workers (clerical workers, care workers, medical assistants, cooking assistants, and others) as study participants. We expected to recruit 40 subjects from each of 20 areas, giving an average recruitment target of four subjects for each dietitian. In each area, eight subjects (four men and four women) were included in each of five 10-year age classes (20–29, 30–39, 40–49, 50–59, and 60–69 years). This target number of participants was determined to meet the original purpose of the survey, which was to assess the sodium and potassium intake of Japanese. In total, 791 participants (395 men and 396 women) were included in the study. The study purpose and protocol were explained to the participants individually by research dietitians, and written informed consent was obtained from each participant. All procedures of the study were approved by the ethics committee of the University of Tokyo Faculty of Medicine (Approval no. 10005).

### 24-hour urine collection

All participants were asked to collect 24-hour urine. Details of the collection method have been reported elsewhere.^21^ Briefly, participants set the starting time of collection and then discarded the first urine at the starting time. All subsequent specimens were collected until the same time as the starting time on the next day. The volume of urine for which collection failed or was forgotten was estimated, recorded, and added to the collected volume. Although approximately 7% of the participants failed to collect urine more than once, the estimated volume of missed urine was less than 1% of the total urine amount from all the participants and similar results were obtained when subjects with incomplete collection were excluded. Total urine volume was then calculated with the adjustment volume based on the collection time. This adjustment method has been validated for measurement of para-aminobenzoic acid.^22^ Urinary iodine content of the collected urine was measured at LSI Medience Corporation (Tokyo, Japan) using an iodine measurement kit (Hitachi Chemical Company Limited, Tokyo, Japan). Details of this microplate method have been described elsewhere.^23^^,^^24^ Briefly, urinary iodine concentration was measured using the Sandell-Kolthoff reaction on microplates and colorimetric measurement. Colorimetric measurement was conducted using the “IMMUNO-MINI NJ-2300” microplate reader (Biotec Company Limited, Tokyo, Japan) at 405 nm. Trueness was assessed by comparing with an inductively coupled plasma mass spectrometry method (within 80–120%), and intra- and inter-assay coefficients of variation (CVs) were calculated for precision (CV <15%).^24^ Creatinine concentration was measured with JCA-BM6050 (JEOL Limited, Tokyo, Japan) using the enzyme method.

### Diet records

For this survey, a 4-day semi-weighed DR was conducted. Half of the study participants were instructed to record a 4-nonconsecutive-day (three working days and one day off) DR, which 392 participants completed. This reduction in the number of subjects recruited for the DR was done in consideration of feasibility and data quality. On average, 20 subjects (generally two men and two women from each of the five 10-year age bands) were selected from each study area. The days before and after a night shift were avoided as recording days. Participants were provided a digital kitchen scale (KD-812WH; Tanita, Tokyo, Japan), measuring spoon, measuring cup, manual for the DR, and recording sheets.

The participants weighed and recorded foods and drinks consumed. They were instructed to weigh ingredients in dishes, prepared dishes after cooking, and drinks using the provided digital scale whenever possible. If subjects ate out and weighing was difficult, they recorded the name of the restaurant and dishes and whether any food was left unconsumed. In addition, the subjects were asked to list the names and manufacturers of all seasonings they routinely used at home on the recording sheet and submit packages of processed foods or snacks with the recording.

The research dietitians explained the recording methods to participants directly and asked them to hand in their recording sheets immediately after recording. They checked the records and directly asked the participants about any missing or unclear information. Recorded food items and weights were then reconfirmed by two research dietitians at the central office of the study. Nutritional value calculations were performed with SAS version 9.3 (SAS Institute, Cary, NC, USA).

Iodine intake was calculated using these DR and a previously developed compensated food composition table.^17^ Iodized salt is not used in Japan.

### Anthropometry data and other variables

Body height was measured in the standing position without shoes to the nearest 0.1 cm, and body weight was measured in light clothing to the nearest 0.1 kg; both measurements were taken by research dietitians or medical workers in the welfare facilities. Body mass index (BMI) was calculated as body weight in kilograms divided by the square of body height in meters. Background information of participants was obtained using a questionnaire.

### Data analysis

Regarding 24-hour urine collection, we confirmed the completeness of collection using the ratio of observed to expected creatinine excretion. Samples with a ratio of <60% or >140% were excluded (*n* = 72), as described previously.^21^ In addition, participants with missing data (*n* = 4) or a history of thyroid disease were excluded (*n* = 2). However, participants taking diuretics or with a history of renal dysfunction were not excluded because participants were workers and were assumed to be sufficiently healthy to work. No subject was excluded from the evaluation of DR because energy intakes calculated from the DR were all within the pre-specified acceptable range of 500–4000 kcal/day. Ultimately, 713 subjects (362 men and 351 women) were included in the analysis of urinary iodine status.

Urine iodine excretion was highly skewed, so it is presented as median and interquartile range (25th and 75th percentiles). Mean values are also shown for comparison with other studies.^2^ Assuming that more than 90% of ingested iodine was excreted in urine,^9^ and using a bioavailability of 92%,^7^ iodine excretion in 24-hour urine was compared with the 2015 DRIs for Japanese (recommended dietary allowance [RDA] of iodine for adults is 130 µg/day, and the UL is 3000 µg/day).^9^ Namely, the percentage of participants whose iodine excretion in 24-hour urine was <119.6 (130*0.92) µg/day or ≥2760 (3000*0.92) µg/day was calculated. Urine iodine concentration was compared with the WHO recommendation for iodine.^3^ Iodine excretion by age group was compared using the Kruskal-Wallis test.

The number of participants with data for both the DR and 24-hour urinary iodine excretion was 357. Correlation between dietary iodine intake and urine iodine excretion was examined using Spearman’s correlation coefficient. Although participants were asked to finish urine collection the day before the first day of diet records, timing overlapped for some participants, while some others recorded their DRs before urine collection. Data from these participants were used to analyze the correlation between iodine intake from the DR and urinary iodine excretion. Correlation coefficients were calculated for participants who (i) recorded the DR and collected urine on the same day; (ii) recorded the DR and collected urine on the same day and recorded the DR 1 day before urine collection; (iii) recorded the DR and collected urine on the same day and recorded the DR 1 day after urine collection; and (iv) for all participants. In the case of (i) to (iii), correlation was calculated for iodine intake in the DR of the overlapping day or the DR 1 day before or after urine collection. Regarding (iv), calculation was done using mean iodine intake over 4 days.

To assess dietary determinants, log-transformed iodine excretion in 24-hour urine was set as the dependent variable, and the intake of food groups, age, sex, and BMI were included as independent variables in linear regression models. Each food group was added into the models individually. Food intake was divided into tertiles after placing participants who did not consume a particular food in the lowest group for that food (ie, the group with zero intake for that food). When less than 50 participants did not consume a certain food, its intake was divided into quartiles, including participants whose intake was zero. The amount of food was added into the models in these divided categories. “Total kelp” was the sum of kelp and kelp for soup stock. Amounts were calculated and expressed as dried weight.^25^ For the calculation of the dried weight of kelp from soup stock, kelp concentration in soup stock for participants who did not record the amount of kelp in soup stock was assumed according to the concentrations in the STFCJ.^15^ “Other seaweed” was the sum of seaweeds other than kelp, calculated as wet weight because not all the dried weights of seaweeds were known.^25^ All analyses were conducted with SAS ver 9.3. Two-sided *P*-values <0.05 were considered statistically significant.

## RESULTS

The study participants are characterized in Table 1. Table 2 shows iodine excretion in the collected 24-hour urine samples, which are expressed as total amount of iodine (µg/24 h), concentration (µg/L), and amount per gram of creatinine excretion (µg/g·Cre). Table 2 also compares iodine excretion and established recommendations (iodine intake determined in 2015 DRIs for Japanese^9^ and iodine concentration in the WHO recommendation^3^). “Excessive” in WHO criteria signifies the amount exceeding the level to prevent and control iodine deficiency.^3^ This definition is different from UL, which is determined to prevent adverse effects of extreme over-intake.^9^ Median iodine excretion was 389 µg/24 h in men and 346 µg/24 h in women. The percentage of participants with excretion below the RDA was 5.5% in men and 9.1% in women, while percentages over the UL were 8.3% in men and 3.7% in women. Only 1% of participants had an iodine concentration <50 µg/L, while 13% were <100 µg/L and 45% were ≥300 µg/L. No significant differences in urinary iodine excretion between research areas or between coastal and mountainous cities were found (data not shown). Iodine excretion by age is given in Table 3. Iodine excretion per day and excretion per gram of creatinine in the older groups were significantly higher than in the younger groups (using the Kruskal-Wallis test and ad hoc Bonferroni test).

**Table 1.** Basic characteristics of study participants

Variables	Total (*n* = 713)		Men (*n* = 362)		Women (*n* = 351)	
		
	Mean	SD	Mean	SD	Mean	SD
Age, years	44.3	13.5	44.4	13.5	44.1	13.6
Body height, cm	163.9	8.4	170.1	5.7	157.4	5.3
Body weight, kg	62.4	12.2	69.0	10.8	55.7	9.5
Body mass index, kg/m^2^	23.1	3.5	23.8	3.4	22.5	3.5

Smoking status	Number	%	Number	%	Number	%
Current smoker	168	23.6	126	34.8	42	11.9
Past smoker	154	21.6	119	32.8	35	9.9
Never smoker	391	54.8	117	32.2	274	78.1
Geographic area^a^						
Hokkaido	29	4.1	15	4.1	14	4.0
Tohoku (Aomori, Iwate, and Yamagata)	71	10.0	38	10.5	33	9.4
Kanto (Ibaraki, Gunma, Saitama, and Kanagawa)	142	19.9	71	19.6	71	20.2
Tokai and Hokuriku (Shizuoka, Niigata, and Toyama)	71	10.0	37	10.2	34	9.7
Kinki (Osaka, Hyogo, and Nara)	113	15.9	57	15.7	56	16.0
Chugoku and Shikoku (Okayama, Hiroshima, Yamaguchi, and Tokushima)	145	20.3	72	19.9	73	20.8
Kyushu (Fukuoka, Saga, Kumamoto, Oita, and Okinawa)	142	19.9	72	19.9	70	20.0
Occupation						
Clerical worker	301	42.2	173	47.8	128	36.5
Care worker	293	41.1	134	37.0	159	45.3
Medical assistant	23	3.2	12	3.3	11	3.1
Cooking assistant	42	5.9	5	1.4	37	10.5
Others	54	7.6	38	10.3	16	4.6
Past history or current treatment						
Hypertension	91	12.8	58	16.0	33	9.4
Hyperlipidemia	59	8.3	28	7.7	31	8.8
Hyperuricemia	16	2.2	15	4.1	1	0.3
Diabetes mellitus	21	2.9	16	4.4	5	1.4
Renal dysfunction, past history	3	0.4	2	0.6	1	0.3

SD, standard deviation.

^a^Participants were recruited in 20 areas throughout Japan; 17 areas were individual prefectures and three areas were combinations of two prefectures each (Aomori and Iwate, Niigata and Toyama, and Fukuoka and Saga). The target number of participants in one area was eight subjects (four men and four women) in each of five 10-year age classes.

**Table 2.** Iodine excretion in 24-hour urine and iodine concentration (µg/L and µg/g·Cre) in 713 Japanese adults

	Mean	SD	Median	25th–75th percentiles	Min	Max	<130 µg/d^a^	≥3000 µg/d^a^	<50 µg/L^b^	<100 µg/L^b^	≥300 µg/L^b^

							Percentage (%)				
Total (*n* = 713)											
Urine volume, mL/24 h	1621	657	1510	1170–2007	384	5040					
I excretion, µg/24 h	897	1564	365	199–996	56	18 161	7.3	6.2	—	—	—
I concentration, µg/L	577	1023	253	131–630	32	12 700	—	—	1.0	13.0	44.7
I excretion, µg/g·Cre	744	1264	301	162–806	42	12 909	—	—	—	—	—
Men (*n* = 362)											
Urine volume, mL/24 h	1714	721	1580	1186–2125	440	5040					
I excretion, µg/24 h	1004	1790	389	206–1061	56	18 161	5.5	8.3	—	—	—
I concentration, µg/L	609	1147	266	133–640	32	12 700			1.4	11.6	45.3
I excretion, µg/g·Cre	674	1183	263	135–706	42	10 211	—	—	—	—	—
Women (*n* = 351)											
Urine volume, mL/24 h	1526	568	1450	1126–1840	384	3460					
I excretion, µg/24 h	787	1285	346	177–948	57	11 844	9.1	3.7	—	—	—
I concentration, µg/L	585	1167	238	126–625	42	8400	—	—	0.6	14.5	44.2
I excretion, µg/g·Cre	816	1340	345	192–932	53	12 909	—	—	—	—	—

Max, maximum; Min, minimum; SD, standard deviation.

^a^The recommended dietary allowance of iodine for adults is 130 µg/day and tolerable upper intake level is 3000 µg/day in Dietary Reference Intakes 2015 for Japanese. Bioavailability of 92% was used. (Percentage of participants whose iodine excretion in 24-hour urine was <130*0.92 or ≥3000*0.92 is shown.)

^b^The World Health Organization (WHO) epidemiologic criteria define urinary iodine concentration of <100 µg/L as “Insufficient” and ≥300 µg/L as “Excessive” for people aged 6 years or older, except for pregnant and lactating women. “Excessive” in WHO criteria means the amount exceeding the level to prevent and control iodine deficiency.

**Table 3.** Iodine excretion in 24-hour urine and iodine concentration (µg/L and µg/g·Cre) by age and sex in 713 Japanese adults

	Age, years										Kruskal-Wallis test

	20–29		30–39		40–49		50–59		60–69		
					
	Median	25th–75th percentiles	Median	25th–75th percentiles	Median	25th–75th percentiles	Median	25th–75th percentiles	Median	25th–75th percentiles	*P* value
Men, *n*	69		79		72		69		73		
Urine, mL	1270	980–1664	1580	1170–1900	1745	1236–2164	1670	1280–2145	1970	1300–2450	<0.0001
I excretion, µg/24 h	306	203–812	326	171–855	312	215–1004	405	203–898	581*	355–1448	0.002
I concentration, µg/L	270	146–610	229	121–482	200	133–640	230	122–560	405	180–760	0.06
I excretion, µg/g·Cre	212	127–628	219	107–546	216	137–600	268	127–661	448*	265–1232	<0.0001

Women, *n*	72		72		71		67		69		
Urine, mL	1216	898–1542	1249	988–1763	1349	1060–1712	1619	1309–2054	1780	1440–2210	<0.0001
I excretion, µg/24 h	275	152–406	249	150–680	298	187–811	489*	293–1308	424*	224–1135	0.0002
I concentration, µg/L	207	128–381	200	115–670	241	138–630	365	172–770	251	126–620	0.17
I excretion, µg/g·Cre	245	162–396	264	136–593	314	191–701	575*	296–1377	493*	251–1279	<0.0001

*Significant difference (*P* < 0.05) with Bonferroni test when compared with the value in the youngest age group (20–29 years).

Correlations between urinary iodine excretion and the intake of iodine calculated from the DR are shown in Table 4. The total amount of urinary iodine excretion per day from urine whose collection overlapped the DR had the highest correlation coefficient with iodine intake from the DR of that overlapped day (“same day” in Table 4; *r* = 0.37, *P* = 0.005). In contrast, data from participants whose DR was completed the day before urine collection (“include before” in Table 4) had a slightly lower correlation coefficient (*r* = 0.31, *P* = 0.01). Finally, the correlation for participants who wrote their DR on the day following urine collection (“include after” in Table 4) and the correlation between the mean 4-day iodine intake and urinary excretion (“all” in Table 4) had was weak (*r* = 0.23, *P* = 0.001 and *r* = 0.15, *P* = 0.004, respectively); however, these correlations were still significant.

**Table 4.** Correlation between iodine intake calculated from the diet records and iodine excretion in Japanese adults

	Same day (*n* = 57)				Include before^a^ (*n* = 63)				Include after^b^ (*n* = 192)				Include before and after (*n* = 198)				All^c^ (*n* = 357)			
	Median	25th–75th percentiles	Spearman		Median	25th–75th percentiles	Spearman		Median	25th–75th percentiles	Spearman		Median	25th–75th percentiles	Spearman		Median	25th–75th percentiles	Spearman	
				
			ρ^d^	*P*			ρ^d^	*P*			ρ^d^	*P*			ρ^d^	*P*			ρ^d^	*P*
Iodine intake,^e^ µg/day	435	106–3569	—	—	435	106–3569	—	—	186	78–877	—	—	191	79–897	—	—	519	176–2034	—	—
Iodine excretion, µg/24 h	464	238–1405	0.37	0.005	443	222–1405	0.31	0.01	420	193–1393	0.23	0.001	410	190–1383	0.21	0.002	388	190–1060	0.15	0.004
Iodine excretion, µg/L	310	140–780	0.34	0.01	281	132–780	0.32	0.01	291	132–765	0.19	0.008	278	130–760	0.19	0.009	262	139–660	0.12	0.03
Iodine excretion, µg/g·Cre	403	218–1080	0.28	0.03	361	196–1080	0.22	0.08	350	169–1071	0.20	0.006	349	168–1061	0.18	0.01	332	157–847	0.14	0.007

^a^Participants who recorded dietary record and urine collection on the same day and recorded dietary record 1 day before urine collection.

^b^Participants who recorded dietary record and urine collection on the same day and recorded dietary record 1 day after urine collection.

^c^Including all the participants who recorded dietary record and collected 24-h urine. Median and 25th and 75th percentiles of iodine intake were calculated from individual mean 4-day iodine intake.

^d^Spearman’s Correlation Coefficient.

^e^Regarding “same day”, “include before” and “include after”, Iodine intake in the dietary record of the overlapped day or the dietary record 1 day on either side of the urine collection day was used, except when correlation was calculated for all participants. For “all”, mean iodine intake over 4 days was used.

Regression models, which included urinary iodine excretion as the dependent variable and dietary factors as the independent variables, are summarized in Table 5. Kelp intake, kelp for soup stock, and fish intake were significant predictors of urinary iodine excretion (*P* = 0.01, *P* = 0.02, and *P* = 0.01, respectively). This significance was not changed when food intakes were entered into the models as continuous values. Other seaweeds were not related to iodine excretion when entered as categorical values (*P* = 0.31) but were significant when continuous values were used (*P* = 0.03). Dairy products were not significantly associated with iodine excretion when entered either as categorical or continuous values. Inclusion of smoking status, research area, and education in the models did not change the results.

**Table 5.** Dietary predictors of iodine excretion in 24-hour urine samples in 357 Japanese adults

Food group	*n*	Contribution to iodine intake, %	I excretion in 24 h, µg/24 h		Kruskal-Wallis test	Linear regression model^a^
Kelp intake, dried weight g/d		57.0	Median	25th–75th percentiles	*P* = 0.01	*P* = 0.01
0	230		357	176–1060		
0.01–0.63	43		410	202–1059		
0.66–1.58	42		313	205–533		
≥1.62	42		507	285–1715		
Kelp for soup stock, dried weight g/d		29.4			*P* = 0.05	*P* = 0.02
0	223		335	175–960		
0.0007–0.13	45		399	239–1293		
0.15–0.75	44		465	205–1513		
≥0.76	45		507	238–1715		
Total kelp intake,^b^ dried weight g/d		86.4				
0	148		317	165–705	*P* = 0.005	*P* = 0.001
0.0007–0.50	71		401	208–1059		
0.50–1.50	69		368	205–994		
≥1.50	69		576	281–1692		
Other seaweeds intake, wet weight g/d		7.3				
0–1.12	89		318	178–1266	*P* = 0.29	*P* = 0.31
1.12–4.28	88		389	189–1056		
4.37–10.5	91		351	203–1297		
≥10.6	89		459	251–1014		
Fish and seafood intake, g/d		1.1				
0–36.0	89		294	171–543	*P* = 0.003	*P* = 0.01
36.2–63.3	89		329	183–898		
64.0–94.0	90		455	208–1448		
≥94.6	89		489	266–1390		
Dairy intake, g/d		0.9				
0–35.8	89		389	182–960	*P* = 0.33	*P* = 0.77
35.8–84.8	89		308	174–1059		
84.8–174.0	90		416	229–1422		
≥175.0	89		402	226–914		

^a^log-transformed 24-h urinary iodine excretion (continuous) as a dependent variable, and intake of each food group (categorical), age (continuous), sex (categorical), and BMI (continuous) as independent variables. Each food group was put into the models individually.

^b^Total kelp is the sum of kelp and kelp for soup stock.

## DISCUSSION

This study is the first to evaluate urinary iodine excretion from 24-hour urine collected from participants throughout Japan. Results showed that iodine intake calculated from a DR was significantly correlated with urinary iodine excretion. Further, the major dietary contributors to urinary iodine excretion in Japan were kelp and fish.

A few papers have reported on urinary iodine excretion in Japanese using 24-hour urine collection, but these studies included a limited number of participants and limited geographic areas. From these previous studies,^2^^,^^26^^,^^27^ mean iodine excretion in 24-hour urine collection from participants who consumed usual diets ranged from 560 µg/24 hours to 3286 µg/24 hours, whereas medians from spot urine ranged from 208 µg/L to 1015 µg/L. Papers that described mean urine iodine as µg/g·Cre reported a range from 660 µg/g·Cre to 3022 µg/g·Cre. Our data are within these ranges. Although iodine intake and excretion are known to show large day-to-day variation^28^^–^^30^ and evaluation from single-day urine might not accurately reflect for the iodine status, the previous estimation of Japanese iodine intake (1.2 mg/day) from household kelp consumption^31^ might be overestimated because calculation from kelp consumption could not consider cooking loss. We were able to determine iodine intake from objective measurements throughout Japan in the present study.

Iodine excretion in 24-hour urine and excretion per gram of creatinine are known to differ by age, with older age groups having higher iodine excretion than younger groups.^32^^,^^33^ Excretion was highest in the 50–60 years or >60 years age groups in these previous studies, and our result is consistent with these findings. Although higher urine volume is known to lead to additional urine iodine excretion,^34^ the significantly higher excretion in the older groups remained even after adjustment for urine volume (data not shown). The non-significant difference in urine concentration between age groups and significantly higher 24-hour excretion of iodine in the older groups might suggest that spot urine is not suitable for the evaluation of 1-day urine iodine excretion. One study concluded that iodine concentration of morning spot urine might not be suitable for evaluating long-term iodine intake because of large intra-individual differences.^30^ Clarifying this point will require a multiple measurement study to compare 24-hour and spot urine samples.

Regarding the timing of iodine intake and excretion, a study in Denmark showed that most iodine absorbed into the body is excreted within 1 day and that there is no significant correlation between iodine intake and excretion on the following day.^28^ In contrast, a Japanese study showed a significant difference between urinary iodine concentration in a group with an iodine-rich diet and a second group without such a diet consumed on the day before urine sampling.^30^ According to a study in France, when additional iodine was ingested in the form of iodized salt at lunch and dinner, the extra amount of iodine was excreted into urine and the excretion on the first day of ingestion was higher compared to the second day.^35^ From these studies, we hypothesized that the correlation between consumed iodine and excretion might be high when the data of iodine intake and excretion were obtained on the same day or urine collection was conducted on the day following the DR. In our protocol, DR recording was started in the morning, whereas urine collection could be started at any time in the day. We saw the highest correlation when the DR and urine collection overlapped. The characteristics of this subgroup were close to those of the other participants who collected urine. Although the sample size was small, this result shows that iodine intake immediately before urine collection is associated with iodine excretion in Japanese. Reported correlation coefficients between iodine intake calculated from the DR and iodine excretion in 24-hour urine were *r* = 0.46^27^ and ρ = 0.79^11^ in Denmark and *r* = 0.52 in Norway.^10^ Considering that our highest correlation coefficient was ρ = 0.37 and that the subgroup with the overlap in DR and urine collection timing had a higher median iodine intake than the group consisting of more participants, estimated iodine intake from DR might differ from true iodine intake of individuals because of the variation in iodine contents in kelp and soup stock. However, a more precise method to estimate iodine intake from DR has not been established. A study with more subjects and multiple measurements of urinary iodine would help to clarify the association.

Regarding dietary contributors, previous studies reported that the contribution of seaweed to iodine intake in Japanese was around 65%; in particular, kelp accounted for around 60%, and soup stock accounted for almost 30%.^17^ These contributions are consistent with our present results and support the significance of the association between kelp, soup stock, and urinary iodine excretion observed in this study. Intake of seaweeds other than kelp was only significantly associated when their weight was used as a continuous value in the regression model. This might have resulted from the difference in the weight of the food groups: the weight of consumed fish was heavier than that of seaweeds, and a difference between seaweed groups could not be detected when categorical values were used. Also, the significant result with continuous values might have been due to a few participants with high seaweed intake. Therefore, the association of intake of seaweeds other than kelp with urinary iodine excretion requires further study.

This study has several limitations. First, the participants were not a random sample. Although licensed medical providers were excluded, most participants were volunteers and might be healthier than the general population. Study participants were co-workers of research dietitians, and the response rate was high (86.7%). We recruited various types of workers in this study and also included some participants in their 60s who were not employed. Nevertheless, height, weight, and smoking status were not largely different from the general population (169.6 cm, 67.8 kg, and 34% current smokers in men; 156.6 cm, 53.7 kg, and 9% current smokers in women).^18^ Further, the survey areas were located throughout Japan, which increases the generalizability of the findings. Second, urinary iodine excretion was measured only once. Because of day-to-day variation, multiple measurement is optimal,^28^ but such repeated measurements were not feasible in this study. Also, the target number of participants for iodine intake was not determined a priori, and it is possible that our sample size was not sufficient to provide statistically representative values of iodine intake. Although further studies are required, this is the first study to show iodine excretion throughout Japan and accordingly serves as a base for future iodine studies. Third, misreporting of dietary intake in the DR and under- or over-estimation of iodine intake might have occurred. Misreporting of food weight was particularly likely when participants were unable to weigh their meals. Soup stock was one of the main sources of iodine intake, but the amount of soup stock and concentration of soup stock source (eg, kelp) sometimes could not be measured when participants ate out. Iodine intake was calculated using values in STFCJ^15^; however, intake might be misestimated, since the iodine content in kelp was not consistent and the amount of kelp used in soup stock was not visible. To minimize misestimation of intakes from eating out, menus from staff canteens and restaurants were obtained.

In conclusion, this study of iodine excretion measured by 24-hour urine collection from several areas of Japan shows that excretion significantly differed among age groups, and that kelp and fish are important determinants of urinary iodine excretion in Japanese. The dietary determinants revealed in this study might be important factors in avoiding the risk of insufficient or excess iodine status and in identifying risk groups with unbalanced dietary habits in the future. Further studies are required to identify threshold values of iodine intake that avoid both excess and deficiency, as well as to explore the association between iodine intake and clinical findings.
